# Comparing methods for aggregating indoor air pollutant concentration over space and time

**DOI:** 10.1088/1742-6596/3140/9/092013

**Published:** 2025-11-01

**Authors:** G Petrou, Y Wang, Z Chalabi, SC Hsu, E Hutchinson, J Milner, P Symonds, M Davies

**Affiliations:** 1 UCL Institute for Environmental Design and Engineering, London, UK; 2 UC Berkeley Centre for the Built Environment, California, USA; 3 UCL Energy Institute, London, UK; 4 LSHTM Department of Public Health, Environments and Society, London, UK

## Abstract

This paper explores the impact of different approaches to aggregating the indoor concentration of fine particulate matter (PM_2.5_) in a case study home. Indoor- and outdoor-sourced PM_2.5_ was modelled in CONTAM-EnergyPlus for a bungalow occupied by a family of four in Plymouth, England. Simulations were conducted assuming energy efficiency levels typical of a 1950s home and following retrofit. Pollutants were modelled at a 5-min temporal resolution in bedrooms, kitchen and living room and aggregated according to four metrics: (i) household arithmetic mean concentration, (ii) household time-weighted mean concentration, (iii) arithmetic mean of individual exposure, and (iv) household arithmetic mean exposure. Comparing the household metrics revealed differences of up to 50.0 % (3.6 μg/m^3^) for the pre-retrofit model, which remained largely consistent following retrofit. When comparing against individual exposure, differences were observed for all three metrics and reached 55.6% (9.1 μg/m^3^) for the pre-retrofit model, and 50.6% (8.7 μg/m^3^) for the post-retrofit model. Approaches (i) and (ii) consistently underpredicted individual exposure. Further, the differences were greatest for the time-weighted mean method, suggesting that taking into consideration the total time that each room is occupied but not when it is occupied will not necessarily provide a more accurate description of occupant exposure compared to the simple arithmetic mean. To better represent individual exposure, data on occupant presence is required.

## 1 Introduction

Exposure to air pollution is thought be linked to 6.7 million premature deaths annually [1]. Recognizing the magnitude of this problem, countries have introduced standards and regulations that have markedly reduced the outdoor concentrations of several pollutants [2], [3]. In comparison, and despite approximately half of pollution-related deaths being linked to household air pollution exposure [1], action to improve indoor air quality lags behind [2].

Part of the challenge in developing standards and regulations to improve indoor air quality is the limited evidence on the health impacts of indoor air pollution exposure [2]. To overcome this issue, studies often rely on evidence established for outdoor air pollution. For example, the relationship between all-cause mortality and long-term average concentration of ambient fine particulate matter (PM_2.5_) has been used in the analysis of indoor exposures [4]. A shortcoming of this approach is the reliance on the assumption of similar toxicity between indoor- and outdoor-generated PM_2.5_. Another shortcoming is the lack of an indoor equivalent to long-term average ambient pollutant concentration. The spatiotemporal variability of indoor pollutant concentration may mean that methods of aggregating outdoor air pollution are not the most appropriate to use.

The simplest aggregation approach would be to place equal weights on each room and estimate the arithmetic mean. To account for the total time that each room is occupied, Shrubsole et al. applied weighting factors to the concentrations of different rooms based on the assumed time spent in each room [5]. Another approach is to consider pollutant concentrations only when rooms are occupied [6], [7]. While it can be argued that the latter two approaches could provide a more representative estimate of occupant pollutant exposure, it is unclear whether this is the case. Motivated by this research gap, this paper investigates whether the aggregation method influences the household estimate of PM_2.5_ and compares it against the occupants’ individual exposure.

## 2 Methods

PM_2.5_, both from indoor and outdoor sources, was modelled in CONTAM-EnergyPlus for an archetypical, naturally ventilated, 1950s bungalow located in Plymouth, England. The following sections summarise the modelling approach, with further detail provided by Wang et al. [8].

### 2.1 Building modelling

Modelling was carried out for the as-built (pre-retrofit) bungalow [8]. Due to the potentially significant impact of home energy efficiency retrofit on indoor air quality [8], [9], modelling was also carried out following retrofit in accordance to Approved Document L 2021 [10]. Pre-retrofit, the house was assumed to have solid walls, solid floor and single-glazed windows. Post-retrofit, internal wall and floor insulation was installed, as well as double glazing. Between May-September, living room and bedroom windows opened during occupied hours if the indoor temperature exceeded 22 °C. During the heating season, only kitchen and bathroom windows opened during cooking and showering. Bathrooms and kitchens were equipped with intermittent extract fans, as detailed in Approved Document F [11]. Two adults and two young children were assumed to live in the bungalow, with occupancy profiles described in previous work [12].

### 2.2 Pollutant Modelling

Simulations were run using a 5-minute timestep, a compromise between computation cost and data resolution, with concentrations reported separately for each room. PM_2.5_ was assumed to be generated indoors during cooking (1.6 mg/min), penetrating from the outdoors (penetration factor of 1 with windows open and 0.8 otherwise [6]), and deposited at a rate of 0.39 h^-1^ [5]. Outdoor PM_2.5_ levels and meteorological data were based on measurements from Plymouth in 2019 [13].

### 2.3 Aggregation metrics

Four approaches to aggregating PM_2.5_ were explored; three approaches provided estimates at the household level, while the fourth resulted in one estimate for each occupant.

#### 2.3.1 Household Arithmetic Mean Concentration (H-AMC)

The concentration (*c*) of each pollutant (*p*) was modelled for each room (*r*) at every timestep (*t*). The household-level arithmetic mean $\left({\bar{c}}_{p}\right)$ for pollutant *p* was calculated as below:

(1) $${\bar{c}}_{p}=\frac{\sum_{r=1}^{R}\;{\bar{c}}_{p,r}}{R}=\frac{\sum_{r=1}^{R}\;\sum_{t=1}^{T}\;c_{p,r,t}}{R\times T},$$

where ${\bar{c}}_{p,r}$ is the arithmetic mean concentration for pollutant *p* in room *r, R* is the total number of rooms, and *T* is the total number of timesteps over one year.

#### 2.3.2 Household Time-Weighted Mean Concentration (H-TWMC)

This time-weighted approach assigns weights to ${\bar{c}}_{p,r}$, based on the total time per room occupancy time:

(2) $${\bar{c}}_{p}^{w}=\sum_{r=1}^{R}\;w_{r}{\bar{c}}_{p,r},$$

where *w*_*r*_ is the weight for room *r*, ${\bar{c}}_{p}^{w}$ is the household-level weighted mean, and $\sum_{r=1}^{R}w_{r}=1$. This approach only considers the total time spent in each room, not which hours of the day are spent in each room. The weights were derived from the assumed occupancy profiles: Kitchen – 0.046, Living room – 0.329, Bedroom 1 – 0.306, Bedroom 2 – 0.160, Bedroom 3 – 0.160.

#### 2.3.3 Individual Arithmetic Mean Exposure (I-AME)

Estimating the individual exposure to indoor pollution (*e*_*p*,*i*,*t*_) requires timeseries data on pollutant concentration in each room and knowledge of each occupant’s room presence within the household:

(3) $$e_{p,i,t}=\sum_{r=1}^{R}\;o_{i,r,t}c_{p,r,t},$$

where *o*_*i*,*r*,*t*_ is an indicator variable that signifies whether individual *i* occupies room *r* for timestep *t*, taking values of 1 or 0. Each individual, if at home, is assumed to only occupy one room for each timestep, thus $\sum_{r=1}^{R}\;o_{i,r,t}=0\;or\;1.$ The arithmetic mean of individual exposure $\left({\bar{e}}_{p,i}\right)$ can be estimated as follows:

(4) $${\bar{e}}_{p,i}=\frac{1}{T}\sum_{t=1}^{T}\;e_{p,i,t}.$$

#### 2.3.4 Household Arithmetic Mean Exposure (H-AME)

This is the arithmetic mean of ${\bar{e}}_{p,i}$:

(5) $${\bar{e}}_{p}^{a}=\frac{1}{I}\sum_{i=1}^{I}\;{\bar{e}}_{p,i},$$

where ***I*** is the total number of occupants in the household.

## 3 Results

### 3.1 Pollutant concentration per room over one year period

**Figure 1:**
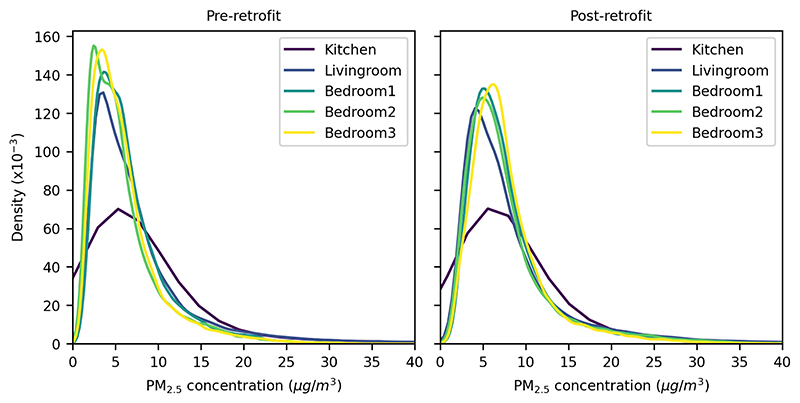
Density plots of pollutant concentration per room. Note: PM_2.5_ concentrations exceed 400 μg/m^3^, but values over 40 μg/m^3^ were not included to improve clarity.

The distribution of PM_2.5_ concentration in each room, pre- and post-retrofit, is characterised by a single peak and a long right tail (Figure 1). As a result, the mean and median values for each room differ by 1.1 μg/m^3^ (18 %; Bedroom 2) to 8.8 μg/m^3^ (140 %; Kitchen) for the pre-retrofit model, with comparable discrepancies for the post-retrofit model. At the household level, the mean and median values differ by 54% for the pre-retrofit model and 45% for the post-retrofit model.

Pre-retrofit, the median values range from 4.7 μg/m^3^ in Bedroom 2 to 6.3 μg/m^3^ in the Kitchen (Table 1). Larger discrepancies between rooms are observed for the mean concentration in each room, ranging from 5.9 μg/m^3^ in Bedroom 2 to 15.1 μg/m^3^ in the Kitchen. Post-retrofit, there is an increase in the mean and median concentration of PM_2.5_ in all rooms. Taking the example of Bedroom 2, the mean and median concentration is 7.9 μg/m^3^ and 6.4 μg/m^3^, corresponding to an increase of 34% and 36%, respectively. Further, post-retrofit the mean concentration of PM_2.5_ is the same, to one decimal place, for all bedrooms.

### 3.2 Comparing different household-level metrics

**Figure 2:**
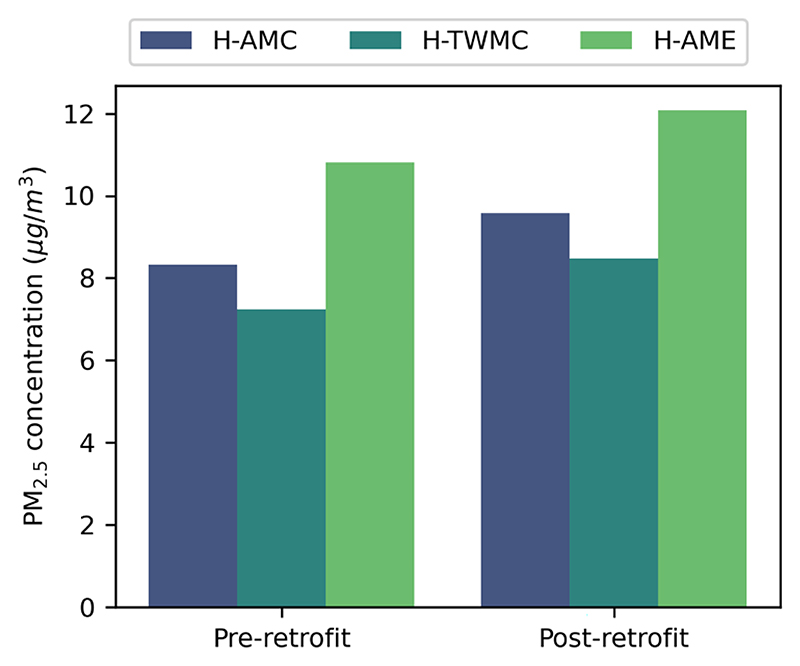
Bar plots comparing household-level pollutant concentration metrics for PM_2.5_.

The comparison between the three household metrics suggests a consistent pattern for the pre- and post-retrofit models for PM_2.5_ (Figure 2): H-TWMC provides the lowest concentration estimate, followed by H-AMC and the H-AME. Focusing on the pre-retrofit model and relative to H-TWMC, H-AMC is 1.1 μg/m^3^ (15.3 %) higher while H-AME is 3.6 μg/m^3^ (50.0 %) higher. Following retrofit, the estimated PM_2.5_ concentration increases by 1.3 μg/m^3^ for all three metrics.

### 3.3 Comparing household-level metrics against individual occupant exposure

**Figure 3:**
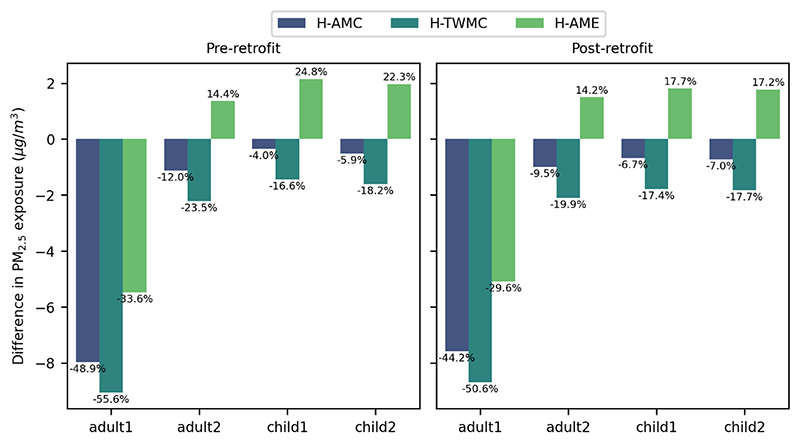
Difference between Individual Arithmetic Mean Exposure and the three household metrics: Household Arithmetic Mean Concentration (H-AMC), Household Time-Weighted Mean Concentration (H-TWMC) and Household Arithmetic Mean Exposure (H-AME).

The absolute and relative differences between I-AME, and the household metrics are presented separately for each occupant in Figure 3. H-AMC and H-TWMC underpredicted PM_2.5_ exposure for all occupants, while H-AME underpredicted the individual exposure for adult 1, but overpredicted the individual exposure for all other occupants. For both retrofit levels, the magnitude of underprediction was smaller for the H-AMC than for the H-TWMC. The discrepancies were greatest for adult 1, reaching 55.6% (9.1 μg/m^3^) for the baseline model, and 50.6% (8.7 μg/m^3^) for the retrofitted model. Comparing the two retrofit levels, the differences tend to be smaller for the more energy efficient model.

## 5 Discussion & Conclusions

Pollutant concentrations in each room were shown to be skewed (Figure 1), resulting in differences in the mean and median concentrations for each room (up to 140 %) and for the household (up to 54 %). An implication of this finding is that the choice between mean and median can have a substantial impact on the reported pollutant concentration, and any subsequent analysis. From a statistical perspective, the use of median may be more appropriate given the skewed pollutant distributions. However, the mean is often preferred in health impact analysis.

Another important finding is the impact that the consideration and potential weighting of pollutant concentrations in different rooms can have on the household estimate of PM_2.5_, with differences in household metrics reaching 50 % for the pre-retrofit model. The per room pollutant concentrations following retrofit are closer in value, resulting in marginally smaller differences in household metrics.

While one could assume that the time-weighted estimate (H-TWMC) would better represent the average household exposure, this was shown not to be the case. Thus, considering the total time spent per room, but not the pollutant concentrations at the specific hours that each room is occupied, does not necessarily improve our estimate of exposure. When compared to the individual mean exposure, no household-level metric consistently resulted in the lowest difference.

Using high spatiotemporal resolution data, this work quantified differences in approaches to aggregating pollutant concentrations. A key limitation of this work is the focus on a single case study that limits the generalisability of our findings. Differences in dwelling characteristics, occupant presence and actions, ventilation levels, pollutant sources and weather could result in pollutant concentrations with different distributions. Nevertheless, the results are illustrative of the relative differences that may arise depending on the choice of aggregation metrics. Furthermore, the co-simulation tool used in this study assumes that each building zone (which corresponds to each room in this case) is well-mixed. This simplifying assumption prevents the estimation of individual inhalation exposure that would be used as the reference point in the analysis. Thus, the Individual Arithmetic Mean Exposure (IAME) was used as reference since it is the closest possible approximation allowed by the modelling framework used.

Nonetheless, this work has revealed numerous findings that can inform modelling and monitoring practice and pave the way for the development of indoor air quality assessment guidelines. A key recommendation of this work is that to comprehensively assess indoor air quality and occupant pollutant exposure, monitoring or modelling of all occupied zones is required, along with occupant presence schedules. In the absence of such data, the metrics derived could substantially under- or over-predict exposure. Further, given the spread in occupant pollutant exposure within the same household, it is important to not only report household estimates, but also individual occupant estimates of pollutant exposure. These recommendations are applicable regardless of the level of home fabric energy efficiency. Future work will expand this analysis to multiple pollutants and consider the effect of different occupant and dwelling characteristics.

## Figures and Tables

**Table 1:** Mean and median PM_2.5_ concentration pre- and post-retrofit for each room and the entire household over one year period.

	Pre-retrofit		Post-retrofit	
	Mean (μg/m^3^)	Median (μg/m^3^)	Mean (μg/m^3^)	Median (μg/m^3^)
Kitchen	15.1	6.3	15.6	7.0
Living R.	7.9	5.8	8.6	6.4
Bedroom 1	6.6	5.5	7.9	6.6
Bedroom 2	5.9	4.7	7.9	6.4
Bedroom 3	6.1	5.0	7.9	6.8
Household	8.3	5.4	9.6	6.6
